# Supplementary material for: Multi-omic machine learning predictor of breast cancer therapy response
Source: Nature. 2021 Dec 7;601(7894):623–9. doi: 10.1038/s41586-021-04278-5 (PMC8791834; doi:10.1038/s41586-021-04278-5)
Supplement: Supplementary file 1 — This file contains Supplementary Methods and Supplementary Tables 1 – 6. [file 41586_2021_4278_MOESM1_ESM.pdf]

nature portfolio

https://doi.org/10.1038/s41586-021-04278-5

Supplementary information

Multi-omic machine learning predictor of breast cancer therapy response

In the format provided by the authors and unedited

Supplementary Methods: Machine Learning Model Specifics

1. Software versions and model parameters

Models were developed in a well-defined environment within a Singularity container (v. 2.4.6-dist). The following software versions were used:

- Python 3.7.4
- Numpy 1.16.4
- Scipy 1.3
- Scikit-learn 0.21.2
- Pandas 0.24.2

To maximise the robustness of the predictions, the models contain two levels of averaging. Firstly, predictions are obtained by averaging three classifier pipelines, as follows:

$$Prob(attaining\ pCR) = \frac{1}{3} \times (Pipeline_{LR}^{HER2+} + Pipeline_{SVC}^{HER2+} + Pipeline_{RF}^{HER2+}),$$

where

$$Pipeline_{Classifier} = \{Coll.Reduction\ (0.8) \Rightarrow Univ.selection\ \Rightarrow Classifier\},$$

with the corresponding hyperparameters listed in Tables 1 and 2. In particular, model hyperparameters that were *fixed a priori* are listed in Table 1, while model hyperparameters that were *optimised* for each of the classifiers using a 5-fold cross-validation setup are listed in Table 3. Once all hyperparameters are set, the model is re-trained on the entire training cohort, and subsequently frozen.

Secondly, to account for possible biases in the optimisation due to the particular cross validation splitting used, we repeated the process explained above 5 times, with 5 different cross-validation splitting seeds (integers from 1 to 5). As a result, we obtain 5 alternative optimised models. The final predictions are the average of the 5.

We trained several versions of the models with increasing numbers of integrated features. Table 2 lists all the feature combinations studied. Each model version was trained independently from all the others. We sometimes refer to the model trained with all available features as the "fully integrated" model.

**Classifier**	**Non-optimised hyperparameters**
Random Forest	bootstrap=True, class_weight=None, criterion='gini', max_leaf_nodes=None, min_impurity_decrease=0.0, min_impurity_split=None, min_samples_leaf=1, min_weight_fraction_leaf=0.0, n_jobs=None, oob_score=False, random_state=1
SVC	cache_size=200, class_weight=None, coef0=0.0, decision_function_shape='ovr', degree=3, max_iter=-1, probability=False, random_state=1, shrinking=True, tol=0.001

Logistic Regression	class_weight=None, dual=False, fit_intercept=True, intercept_scaling=1, max_iter=10000, multi_class='warn', n_jobs=None, penalty='elasticnet', random_state=1, solver='saga', tol=0.0001, warm_start=False

Table 1. List of non-optimised classifier hyperparameters.

Model num.	Clinical	DNA	RNA	Digital Pathology	Treatment
**1**	x				
**2**	x	x			
**3**	x		x		
**4**	x	x	x		
**5**	x	x	x	x	
**6**	x	x	x	x	x

Table 2. List of all the trained models depending on features used.

2. Features selected after dimensionality reduction steps

The modelling pipeline includes two steps of dimensionality reduction before classification, namely collinearity reduction and univariable feature selection. Collinearity reduction acts by removing features correlated by over 0.8 (Spearman correlation). The univariable feature selection step acts by keeping only the *k* best features in terms of their predictive power. This number *k* is set during the optimization process.

The lists of features that result from the two-step dimensionality reduction process in each of the model pipelines are displayed in Tables 4 to 9.

3. Logistic regression coefficients

We estimated feature importances by dropping one feature at a time and re-calculating the cross-validation AUC, as explained in the main body of the manuscript. This approach is general enough to be applicable to all algorithms.

To provide further insights into the features driving the prediction, Tables 10 to 15 contain the logistic regression coefficients for each of the models, as well as the mean and standard deviation values used to z-score normalise the features.

		Logistic Regression pipeline			Random Forest pipeline					Support Vector Machine pipeline			
Seed	Features	K Best	C	L1 ratio	K Best	Max. depth	Max. features	Min. samples split	Num. estimators	K Best	C	Gamma	Kernel
1	Clinical	6	0.045	0.1	6	3	0.2	6	50	4	0.086	6.02E-08	sigmoid
2	Clinical	4	0.028	0.1	4	None	0.7	15	5	4	0.017	0.001124	linear
3	Clinical	6	0.073	0.1	6	3	0.7	10	10	4	0.034	6.02E-08	rbf
4	Clinical	4	0.045	0.2	4	3	0.05	12	10	4	0.276	8.90E-09	rbf
5	Clinical	5	0.017	0.1	5	None	0.2	15	10	4	60.209	5.36E-07	rbf
1	Clin. + DNA	14	0.117	0.1	14	None	0.1	12	50	13	1.421	1.87E-05	rbf
2	Clin. + DNA	7	0.045	0.1	all	None	0.1	12	50	8	0.137	0.001941	sigmoid
3	Clin. + DNA	14	0.788	0.8	14	3	0.1	15	50	14	11.690	0.004406	rbf
4	Clin. + DNA	all	0.045	0.1	12	None	0.2	15	10	11	0.005	2.27E-09	rbf
5	Clin. + DNA	all	0.489	0.2	all	3	0.2	10	10	all	11.690	0.004406	rbf
1	Clin. + RNA	13	0.073	0.1	13	3	0.05	2	50	10	14.774	1.17E-08	rbf
2	Clin. + RNA	all	0.117	0.1	3	None	0.05	6	10	10	1.421	1.00E-09	rbf
3	Clin. + RNA	13	0.073	0.1	all	None	0.05	10	10	13	18.672	1.73E-09	rbf
4	Clin. + RNA	10	0.028	0.1	13	None	0.05	10	25	11	0.108	4.58E-08	rbf
5	Clin. + RNA	13	0.028	0.1	13	3	0.1	3	50	9	0.003	0.001124	rbf
1	Clin. + DNA + RNA	20	0.189	0.1	all	None	0.05	2	100	all	1000.000	1.31E-09	rbf
2	Clin. + DNA + RNA	20	0.045	0.1	18	3	0.05	12	25	14	0.108	6.02E-08	rbf
3	Clin. + DNA + RNA	15	0.189	0.1	all	None	0.05	6	100	13	245.375	6.02E-08	rbf
4	Clin. + DNA + RNA	all	0.189	0.1	all	3	0.05	10	10	all	2.270	1.00E-09	rbf
5	Clin. + DNA + RNA	all	0.045	0.1	all	None	0.2	15	10	14	5.790	1.80E-07	rbf
1	Clin. + DNA + RNA + DigPath	21	0.189	0.2	19	None	0.2	6	10	15	0.013	5.15E-09	rbf
2	Clin. + DNA + RNA + DigPath	all	0.045	0.1	all	None	0.05	3	50	14	1.124	1.54E-08	rbf
3	Clin. + DNA + RNA + DigPath	18	0.304	0.6	10	3	0.7	3	25	16	18.672	6.26E-06	rbf
4	Clin. + DNA + RNA + DigPath	all	2.043	0.1	9	3	0.7	10	50	20	76.095	1.00E-09	rbf
5	Clin. + DNA + RNA + DigPath	all	0.073	0.1	21	None	0.05	6	50	16	0.002	0.001941	rbf
1	Clin. + DNA + RNA + DigPath + Chemo	21	0.304	0.3	all	None	0.05	6	100	15	0.013	5.15E-09	rbf
2	Clin. + DNA + RNA + DigPath + Chemo	22	0.045	0.1	25	None	0.05	12	25	14	3.625	1.08E-05	rbf
3	Clin. + DNA + RNA + DigPath + Chemo	15	0.073	0.1	all	None	0.7	2	25	16	18.672	6.26E-06	rbf
4	Clin. + DNA + RNA + DigPath + Chemo	23	1.269	0.1	all	None	0.1	6	25	24	153.617	1.00E-09	rbf
5	Clin. + DNA + RNA + DigPath + Chemo	23	0.073	0.3	all	3	0.7	6	10	all	0.003	1.00E-09	rbf

Table 3. Optimised hyperparameters used in each of the three model pipelines (rbf: radial basis function)

	Logistic Regression					Random Forest					SVC				
Seeds	**1**	**2**	**3**	**4**	**5**	**1**	**2**	**3**	**4**	**5**	**1**	**2**	**3**	**4**	**5**
**Tumour size**	x		x			x		x							
**Lymph node involvement**	x	x	x	x	x	x	x	x	x	x	x	x	x	x	x
**Age at diagnosis**	x	x	x	x	x	x	x	x	x	x	x	x	x	x	x
**Histological subtype**	x		x		x	x		x		x					
**ER status**	x	x	x	x	x	x	x	x	x	x	x	x	x	x	x
**Histological grade**	x	x	x	x	x	x	x	x	x	x	x	x	x	x	X

Table 4. Features selected after the dimensionality reduction steps, for the clinical model.

	Logistic Regression					Random Forest					SVC				
Seeds	**1**	**2**	**3**	**4**	**5**	**1**	**2**	**3**	**4**	**5**	**1**	**2**	**3**	**4**	**5**
**Tumour size**	x		x	x	x	x	x	x		x	x		x		x
**Lymph node involvement**	x	x	x	x	x	x	x	x	x	x	x	x	x	x	x
**Age at diagnosis**	x	x	x	x	x	x	x	x	x	x	x	x	x	x	x
**Histological subtype**	x		x	x	x	x	x	x	x	x	x		x	x	x
**HER2 status**	x		x	x	x	x	x	x		x			x		x
**ER status**	x	x	x	x	x	x	x	x	x	x	x	x	x	x	x
**Histological grade**	x	x	x	x	x	x	x	x	x	x	x	x	x	x	x
**TMB**	x	x	x	x	x	x	x	x	x	x	x	x	x	x	x
***PIK3CA* mutation status**	x		x	x	x	x	x	x	x	x	x		x	x	x
***TP53* mutation status**	x		x	x	x	x	x	x	x	x	x	x	x	x	x
**Chromosomal instability**	x	x	x	x	x	x	x	x	x	x	x	x	x	x	x
**HRD score**	x	x	x	x	x	x	x	x	x	x	x	x	x	x	x
**Neoantigen burden**	x		x	x	x	x	x	x	x	x	x		x		x
**LOH HLA**	x		x	x	x	x	x	x	x	x	x		x	x	x

Table 5. Features selected after the dimensionality reduction steps, for the clinical+DNA model.

	Logistic Regression					Random Forest					SVC				
Seeds	**1**	**2**	**3**	**4**	**5**	**1**	**2**	**3**	**4**	**5**	**1**	**2**	**3**	**4**	**5**
**Tumour size**	x	x	x		x	x		x	x	x			x		
**Lymph node involvement**	x	x	x	x	x	x		x	x	x	x	x	x	x	x
**Age at diagnosis**	x	x	x	x	x	x		x	x	x	x	x	x	x	x
**Histological subtype**	x	x	x		x	x		x	x	x			x	x	
**HER2 status**	x	x	x		x	x		x	x	x			x		
**ER status**	x	x	x	x	x	x		x	x	x	x	x	x	x	x
***PGR* expression**	x	x	x	x	x	x	x	x	x	x	x	x	x	x	x
***ESR1* expression**	x	x	x	x	x	x	x	x	x	x	x	x	x	x	x
**Histological grade**	x	x	x	x	x	x		x	x	x	x	x	x	x	x
**Taxane score**	x	x	x	x	x	x	x	x	x	x	x	x	x	x	x
**STAT1 score**	x	x	x	x	x	x		x	x	x	x	x	x	x	x
**T-cell dysfunction score**		x						x							
**T-cell exclusion score**	x	x	x	x	x	x		x	x	x	x	x	x	x	
**Mast cell score**	x	x	x	x	x	x		x	x	x	x	x	x	x	x

Table 6. Features selected after the dimensionality reduction steps, for the clinical+RNA model.

	Logistic Regression					Random Forest					SVC				
Seeds	**1**	**2**	**3**	**4**	**5**	**1**	**2**	**3**	**4**	**5**	**1**	**2**	**3**	**4**	**5**
**Tumour size**	x	x		x	x	x		x	x	x	x			x	
**Lymph node involvement**	x	x	x	x	x	x	x	x	x	x	x	x	x	x	x
**Age at diagnosis**	x	x	x	x	x	x	x	x	x	x	x	x	x	x	x
**Histological subtype**	x	x		x	x	x	x	x	x	x	x			x	
**HER2 status**	x	x		x	x	x		x	x	x	x			x	
**ER status**	x	x	x	x	x	x	x	x	x	x	x	x	x	x	x
***PGR* expression**	x	x	x	x	x	x	x	x	x	x	x	x	x	x	x
***ESR1* expression**	x	x	x	x	x	x	x	x	x	x	x	x	x	x	x
**Histological grade**	x	x	x	x	x	x	x	x	x	x	x	x	x	x	x
**Taxane score**	x	x	x	x	x	x	x	x	x	x	x	x	x	x	x
**TMB**	x	x	x	x	x	x	x	x	x	x	x	x	x	x	x
***PIK3CA* mutation status**	x	x	x	x	x	x	x	x	x	x	x			x	
***TP53* mutation status**	x	x	x	x	x	x	x	x	x	x	x	x		x	x
**Chromosomal instability**	x	x	x	x	x	x	x	x	x	x	x	x	x	x	x
**HRD score**	x	x	x	x	x	x	x	x	x	x	x	x	x	x	x
**Neoantigen burden**	x	x		x	x	x	x	x	x	x	x			x	
**STAT1 score**	x	x	x	x	x	x	x	x	x	x	x	x	x	x	x
**LOH HLA**	x	x		x	x	x	x	x	x	x	x			x	
**T-cell dysfunction score**				x	x	x		x	x	x	x			x	
**T-cell exclusion score**	x	x	x	x	x	x	x	x	x	x	x	x	x	x	x
**Mast cell score**	x	x	x	x	x	x	x	x	x	x	x	x	x	x	x

Table 7. Features selected after the dimensionality reduction steps, for the clinical+DNA+RNA model.

	Logistic Regression					Random Forest					SVC				
Seeds	**1**	**2**	**3**	**4**	**5**	**1**	**2**	**3**	**4**	**5**	**1**	**2**	**3**	**4**	**5**
**Tumour size**	x	x		x	x		x			x				x	
**Lymph node involvement**	x	x	x	x	x	x	x			x	x	x	x	x	x
**Age at diagnosis**	x	x	x	x	x	x	x			x	x	x	x	x	x
**Histological subtype**	x	x	x	x	x	x	x			x				x	
**HER2 status**	x	x		x	x		x			x					
**ER status**	x	x	x	x	x	x	x	x	x	x	x	x	x	x	x
***PGR* expression**	x	x	x	x	x	x	x	x	x	x	x	x	x	x	x
***ESR1* expression**	x	x	x	x	x	x	x	x	x	x	x	x	x	x	x
**Histological grade**	x	x	x	x	x	x	x	x	x	x	x	x	x	x	x
**Taxane score**	x	x	x	x	x	x	x	x	x	x	x	x	x	x	x
**TMB**	x	x	x	x	x	x	x			x	x	x	x	x	x
***PIK3CA* mutation status**	x	x	x	x	x	x	x			x			x	x	x
***TP53* mutation status**	x	x	x	x	x	x	x			x	x		x	x	x
**Chromosomal instability**	x	x	x	x	x	x	x	x	x	x	x	x	x	x	x

**HRD score**	x	x	x	x	x	x	x	x	x	x	x	x	x	x	x
**Neoantigen burden**	x	x		x	x	x	x			x				x	
**STAT1 score**	x	x	x	x	x	x	x	x		x	x	x	x	x	x
**Lymphocyte density**	x	x	x	x	x	x	x	x	x	x	x	x	x	x	x
**LOH HLA**	x	x	x	x	x	x	x			x				x	
**T-cell dysfunction score**		x		x	x		x								
**T-Cell exclusion score**	x	x	x	x	x	x	x			x	x	x	x	x	x
**Mast cell score**	x	x	x	x	x	x	x	x	x	x	x	x	x	x	x

Table 8. Features selected after the dimensionality reduction steps, for the clinical+DNA+RNA+Digital Pathology model.

	Logistic Regression					Random Forest					SVC				
Seeds	**1**	**2**	**3**	**4**	**5**	**1**	**2**	**3**	**4**	**5**	**1**	**2**	**3**	**4**	**5**
**Tumour size**	x	x		x	x	x	x	x	x	x				x	x
**Lymph node involvement**	x	x	x	x	x	x	x	x	x	x	x	x	x	x	x
**Age at diagnosis**	x	x	x	x	x	x	x	x	x	x	x	x	x	x	x
**Histological subtype**	x	x		x	x	x	x	x	x	x				x	x
**HER2 status**	x	x		x	x	x	x	x	x	x				x	x
**ER status**	x	x	x	x	x	x	x	x	x	x	x	x	x	x	x
***PGR* expression**	x	x	x	x	x	x	x	x	x	x	x	x	x	x	x
***ESR1* expression**	x	x	x	x	x	x	x	x	x	x	x	x	x	x	x
**Histological grade**	x	x	x	x	x	x	x	x	x	x	x	x	x	x	x
**Taxane score**	x	x	x	x	x	x	x	x	x	x	x	x	x	x	x
**TMB**	x	x	x	x	x	x	x	x	x	x	x	x	x	x	x
***PIK3CA* mutation status**	x	x		x	x	x	x	x	x	x			x	x	x
***TP53* mutation status**	x	x	x	x	x	x	x	x	x	x	x		x	x	x
**Chromosomal instability**	x	x	x	x	x	x	x	x	x	x	x	x	x	x	x
**HRD score**	x	x	x	x	x	x	x	x	x	x	x	x	x	x	x
**Neoantigen burden**	x	x		x	x	x	x	x	x	x				x	x
**STAT1 score**	x	x	x	x	x	x	x	x	x	x	x	x	x	x	x
**Lymphocyte density**	x	x	x	x	x	x	x	x	x	x	x	x	x	x	x
**LOH HLA**	x	x		x	x	x	x	x	x	x				x	x
**T-cell dysfunction score**						x		x	x	x					x
**T-Cell exclusion score**	x	x	x	x	x	x	x	x	x	x	x	x	x	x	x
**Mast cell score**	x	x	x	x	x	x	x	x	x	x	x	x	x	x	x
**Anthracycline therapy**				x	x	x	x	x	x	x				x	x
**Taxane first**						x	x	x	x	x					x
**Taxane second**						x	x	x	x	x				x	x
**Number of chemo cycles**		x		x	x	x	x	x	x	x				x	x

Table 9. Features selected after the dimensionality reduction steps, for the clinical+DNA+RNA+Digital Pathology+Treatment model.

Seeds	1	2	3	4	5	Mean	Std. Dev.
**Age at diagnosis**	-0.152	0.171	-0.177	0.199	-0.077	49.973	116.258
**Histological subtype**	0.227		0.281		0.109	0.891	0.097
**ER status**	-0.223	-0.155	-0.293	-0.188	-0.089	0.320	0.898
**Histological grade**	0.052	-0.236	0.078	-0.288	0.000	2.612	0.237

Table 10. Logistic regression coefficients and scores used to z-score normalize the features, for the model including clinical features.

Seeds	1	2	3	4	5	Mean	Std. Dev.
**Tumour size**	-0.184		-0.244	-0.102	-0.250	45.850	689.365
**Lymph node involvement**	-0.149	0.132	-0.162	-0.124	-0.170	0.048	0.998
**Age at diagnosis**	0.173	0.146	0.188	0.136	0.210	49.973	116.258
**Histological subtype**	-0.091		-0.372	0.000	-0.382	0.891	0.097
**HER2 status**	0.330		0.485	0.229	0.470	-0.252	0.937
**ER status**	0.102	-0.265	0.110	0.054	0.136	0.320	0.898
**Histological grade**	0.282	0.153	0.519	0.127	0.509	2.612	0.237
**TMB**	-0.089	0.220	-0.075	-0.050	-0.093	96.340	6371.299
***PIK3CA* mutation status**	0.044		0.090	0.009	0.120	0.259	0.192
***TP53* mutation status**	0.202		0.292	0.129	0.273	0.578	0.244
**HRD score**	-0.382	-0.231	-0.606	-0.225	-0.588	26.340	234.878
**Neoantigen burden**	0.212		0.261	0.161	0.291	24.619	567.270
**LOH HLA**	-0.080		-0.073	-0.046	-0.099	0.177	0.146

Table 11. Logistic regression coefficients and scores used to z-score normalize the features, for the model including clinical+DNA features.

Seeds	1	2	3	4	5	Mean	Std. Dev.
**Tumour size**	-0.141	0.000	-0.141		-0.075	45.850	689.365
**Lymph node involvement**	0.087	0.081	0.087	0.150	0.069	0.048	0.998
**Age at diagnosis**	-0.301	-0.352	-0.301	-0.076	-0.199	49.973	116.258
**Histological subtype**	0.177	0.171	0.177		0.149	0.891	0.097
**ER status**	-0.134	-0.150	-0.134	0.070	-0.086	0.320	0.898
***PGR* expression**	0.111	0.163	0.111	-0.086	0.028	0.973	7.114
***ESR1* expression**	-0.315	-0.400	-0.315	-0.151	-0.152	3.628	7.968
**Histological grade**	0.041	0.027	0.041	-0.199	0.047	2.612	0.237
**Taxane score**	-0.137	-0.157	-0.137	-0.092	-0.092	-0.816	1.902
**STAT1 score**	-0.039	-0.058	-0.039	-0.230	0.000	21230.641	3702977.558
**Mast cell score**	0.184	0.252	0.184	0.050	0.059	3.040	2.968

Table 12. Logistic regression coefficients and scores used to z-score normalize the features, for the model including clinical+RNA features.

Seeds	1	2	3	4	5	Mean	Std. Dev.
**Tumour size**	-0.232	-0.108		0.012	0.000	45.850	689.365
**Lymph node involvement**	-0.025	-0.009	0.015	-0.024	-0.009	0.048	0.998
**Age at diagnosis**	-0.259	0.000	-0.391	-0.259	0.000	49.973	116.258
**HER2 status**	-0.198	-0.142		-0.229	-0.108	-0.252	0.937
**ER status**	0.000	0.026	-0.001	0.000	0.027	0.320	0.898
***PGR* expression**	0.180	0.061	-0.166	0.179	0.061	0.973	7.114
***ESR1* expression**	-0.499	-0.228	-0.471	-0.498	-0.228	3.628	7.968
**Histological grade**	0.173	0.077	0.102	0.172	0.077	2.612	0.237
**Taxane score**	-0.255	-0.117	-0.248	-0.256	-0.117	-0.816	1.902
**TMB**	-0.202	-0.095	0.135	-0.203	-0.095	96.340	6371.299
***PIK3CA* mutation status**	0.014	0.060	-0.116	0.011	0.060	0.259	0.192
***TP53* mutation status**	0.255	0.134	-0.265	0.256	0.134	0.578	0.244
**HRD score**	-0.190	-0.109	0.197	-0.190	-0.109	26.340	234.878
**Neoantigen burden**	0.100	0.129		0.102	0.129	24.619	567.270
**STAT1 score**	0.005	0.000	0.177	0.006	0.000	21230.641	3702977.558
**T-cell dysfunction score**				-0.203	-0.142	-0.169	0.818
**T-Cell exclusion score**	0.140	0.078	-0.417	0.139	0.078	-0.206	0.941
**Mast cell score**	0.399	0.136	0.051	0.400	0.136	3.040	2.968

Table 13. Logistic regression coefficients and scores used to z-score normalize the features, for the model including clinical+DNA+RNA features.

Seeds	1	2	3	4	5	Mean	Std. Dev.
**Tumour size**	-0.274	-0.065		-0.284	-0.080	45.850	689.365
**Lymph node involvement**	-0.208	0.000	0.155	-0.735	-0.058	0.048	0.998
**Age at diagnosis**	0.108	0.073	0.000	0.296	0.090	49.973	116.258
**Histological subtype**	0.061	0.118	0.476	-0.202	0.112	0.891	0.097
**HER2 status**	0.448	-0.158		-0.406	-0.194	-0.252	0.937
**ER status**	0.000	-0.009	0.000	0.000	-0.016	0.320	0.898
***PGR* expression**	0.115	0.050	0.075	0.268	0.081	0.973	7.114
***ESR1* expression**	-0.426	-0.211	-0.383	-0.828	-0.289	3.628	7.968
**Histological grade**	0.000	0.000	0.006	0.058	0.000	2.612	0.237
**TMB**	0.230	0.129	-0.547	0.480	0.164	96.340	6371.299
***TP53* mutation status**	0.000	0.020	0.000	-0.350	0.000	0.578	0.244
**Chromosomal instability**	0.120	0.066	-0.063	0.197	0.091	0.354	0.029
**HRD score**	-0.165	-0.105	0.000	-0.252	-0.130	26.340	234.878
**Neoantigen burden**	-0.041	0.000		-0.284	0.000	24.619	567.270
**STAT1 score**	0.000	0.000	-0.176	0.019	-0.004	21230.641	3702977.558
**T-cell dysfunction score**		0.230		0.972	0.301	-0.169	0.818
**Mast cell score**	0.400	0.145	-0.191	0.857	0.231	3.040	2.968

Table 14. Logistic regression coefficients and scores used to z-score normalize the features, for the model including clinical+DNA+RNA+Digital Pathology features.

Seeds	**1**	**2**	**3**	**4**	**5**	**Mean**	**Std. Dev.**
**Tumour size**	-0.326	-0.155		-0.390	-0.168	45.850	689.365
**Age at diagnosis**	0.116	0.070	0.121	0.220	0.032	49.973	116.258
**Histological subtype**	0.020	0.117		-0.055	0.097	0.891	0.097
**HER2 status**	0.527	0.231		0.912	0.280	-0.252	0.937
**ER status**	0.000	-0.007	0.062	0.066	0.000	0.320	0.898
***PGR* expression**	0.128	0.051	-0.128	0.202	0.001	0.973	7.114
***ESR1* expression**	-0.490	-0.208	-0.287	-0.727	-0.212	3.628	7.968
**Histological grade**	0.000	0.000	0.003	0.058	0.000	2.612	0.237
**TMB**	0.266	0.131	-0.161	0.499	0.128	96.340	6371.299
***TP53* mutation status**	0.000	0.019	0.294	-0.342	0.000	0.578	0.244
**Chromosomal instability**	0.132	0.065	0.133	0.141	0.034	0.354	0.029
**HRD score**	-0.176	-0.108	0.103	-0.273	-0.087	26.340	234.878
**Neoantigen burden**	-0.006	-0.063		-0.130	-0.016	24.619	567.270
**STAT1 score**	0.000	0.000	-0.264	0.055	0.000	21230.641	3702977.558
**Mast cell score**	0.486	0.145	0.026	0.804	0.135	3.040	2.968
**Anthracycline therapy**				-0.234	0.000	0.918	0.075
**Number of chemo cycles**		0.039		0.373	0.000	5.925	0.450

Table 15. Logistic regression coefficients and scores used to z-score normalize the features, for the model including clinical+DNA+RNA+Digital Pathology+Treatment features.
