# Supplementary material for: Multi-omic machine learning predictor of breast cancer therapy response
Source: Nature. 2021 Dec 7;601(7894):623–9. doi: 10.1038/s41586-021-04278-5 (PMC8791834; doi:10.1038/s41586-021-04278-5)
Supplement: Supplementary file 2 — Reporting Summary [file 41586_2021_4278_MOESM2_ESM.pdf]

nature portfolio

Corresponding author(s): Carlos Caldas

Last updated by author(s): Oct 22, 2021

Reporting Summary

Nature Portfolio wishes to improve the reproducibility of the work that we publish. This form provides structure for consistency and transparency in reporting. For further information on Nature Portfolio policies, see our Editorial Policies and the Editorial Policy Checklist.

Statistics

For all statistical analyses, confirm that the following items are present in the figure legend, table legend, main text, or Methods section.

n/a	Confirmed	
☐	☒	The exact sample size (n) for each experimental group/condition, given as a discrete number and unit of measurement
☐	☒	A statement on whether measurements were taken from distinct samples or whether the same sample was measured repeatedly
☐	☒	The statistical test(s) used AND whether they are one- or two-sided<br>*Only common tests should be described solely by name; describe more complex techniques in the Methods section.*
☐	☒	A description of all covariates tested
☐	☒	A description of any assumptions or corrections, such as tests of normality and adjustment for multiple comparisons
☐	☒	A full description of the statistical parameters including central tendency (e.g. means) or other basic estimates (e.g. regression coefficient) AND variation (e.g. standard deviation) or associated estimates of uncertainty (e.g. confidence intervals)
☐	☒	For null hypothesis testing, the test statistic (e.g. F, t, r) with confidence intervals, effect sizes, degrees of freedom and P value noted<br>*Give P values as exact values whenever suitable.*
☒	☐	For Bayesian analysis, information on the choice of priors and Markov chain Monte Carlo settings
☒	☐	For hierarchical and complex designs, identification of the appropriate level for tests and full reporting of outcomes
☒	☐	Estimates of effect sizes (e.g. Cohen's d, Pearson's r), indicating how they were calculated

Our web collection on statistics for biologists contains articles on many of the points above.

Software and code

Policy information about availability of computer code

Data collection

Clinical data was collected in Microsoft Excel (as part of the office 365 suite) by data managers, and then converted into R objects using the R statistical framework (v 4.0.3)

Data analysis

List of software used:

ANNOVAR: version 599af129dbcfd4e85a2da9832c4ae59898e2f3a9
ASCAT: version 2.5.1
bcl2fastq2: version 2.17
CellExtractor: version v1.0
Ensembl Variant Effect Predictor: version 87
FastQC: version 0.11.7
Genome Analysis Toolkit (GATK): version 4.1.4. Tools used: BaseRecalibrator, CreateSomaticPanelOfNormals, FilterMutectCalls, HaplotypeCaller, IndelRealigner, Mutect2, RealignerTargetCreator, SplitNCigarReads, VariantRecalibrator
HTSeq: version 0.6.1p1
LOHHLA: https://bitbucket.org/mcgranahanlab/lohhla/src/master/ commit 9d58c99
Microsoft Excel: office 365 version
Novoalign and Novosort: version 3.2.13
NetMHC: version 4
NetMHCPan: version 3
Picard: version 2.17.0. Tools used: CalculateHSMetrics, MarkDuplicates
PickPocket: version 1.1
Polysolver: version 4

pVAC-tools: version 1.5.4
Singularity: version 2.4.6-dist
STAR: version 2.5.2b
TIDE: http://tide.dfci.harvard.edu

R version 4.0.3 and associated packages:
• DeconstructSigs: version 1.8
• DNAcopy: version 1.60
• edgeR: version 3.32.1
• GSVA: version 1.34
• Hmisc version 4.4
• iC10: version 1.5
• MASS: version 7.3-54
• MCPcounter: version 1.2.0
• pheatmap: version 1.0.12
• QDNAseq: version 1.24
• ReactomePA: version 1.34
• scarHRD: version 0.1.1
• vcd: version 1.4-7

Python version 3.7.4 and associated packages:
• Numpy: version 1.16.4
• Scipy: version 1.3
• Scikit-learn: version 0.21.2
• Pandas: version 0.24.2

For manuscripts utilizing custom algorithms or software that are central to the research but not yet described in published literature, software must be made available to editors and reviewers. We strongly encourage code deposition in a community repository (e.g. GitHub). See the Nature Portfolio guidelines for submitting code & software for further information.

Data

Policy information about availability of data

All manuscripts must include a data availability statement. This statement should provide the following information, where applicable:

- Accession codes, unique identifiers, or web links for publicly available datasets
- A description of any restrictions on data availability
- For clinical datasets or third party data, please ensure that the statement adheres to our policy

DNA and RNA sequence data have been deposited at the European Genome-phenome Archive (EGA), which is hosted by the EBI and the CRG, under accession number EGAS00001004582 (https://ega-archive.org).

Individual raw data sets are available in Supplementary Tables 1–4.

The R and Python source code used to run the analyses described in the manuscript and to generate all figures is available at: https://github.com/cclab-brca/neoadjuvant-therapy-response-predictor

The following gene sets are referenced within the manuscript:

1. Molecular Signatures Database (MSigDB) Hallmarks gene set (version 6.1). Downloaded from: https://www.gsea-msigdb.org/gsea/msigdb/
2. Genomic Grade Index (GGI) gene set. Reference: Sotiriou, C. et al. Gene expression profiling in breast cancer: understanding the molecular basis of histologic grade to improve prognosis. J. Natl. Cancer Inst. 98, 262–72 (2006).
3. Core Embryonic stem cell (ESC)-like module. Reference: Wong, D. J. et al. Module map of stem cell genes guides creation of epithelial cancer stem cells. Cell Stem Cell 2, 333–44 (2008).
4. STAT1 immune signature. Reference: Desmedt, C. et al. Biological processes associated with breast cancer clinical outcome depend on the molecular subtypes. Clin. Cancer Res. 14, 5158–65 (2008).
5. Paclitaxel response metagene. Reference: Juul, N. et al. Assessment of an RNA interference screen-derived mitotic and ceramide pathway metagene as a predictor of response to neoadjuvant paclitaxel for primary triple-negative breast cancer: a retrospective analysis of five clinical trials. Lancet. Oncol. 11, 358–65 (2010).
6. Cytolytic activity (CYT) score. Reference: Rooney, M. S., Shukla, S. A., Wu, C. J., Getz, G. & Hacohen, N. Molecular and genetic properties of tumors associated with local immune cytolytic activity. Cell 160, 48–61 (2015).
7. Danaher immune gene sets. Reference: Danaher, P. et al. Gene expression markers of Tumor Infiltrating Leukocytes. J. Immunother. Cancer 5, 18 (2017).
8. Immunoscore gene sets. Reference: Charoentong, P. et al. Pan-cancer Immunogenomic Analyses Reveal Genotype-Immunophenotype Relationships and Predictors of Response to Checkpoint Blockade. Cell Rep. 18, 248–262 (2017).

Field-specific reporting

Please select the one below that is the best fit for your research. If you are not sure, read the appropriate sections before making your selection.

☒ Life sciences ☐ Behavioural & social sciences ☐ Ecological, evolutionary & environmental sciences

For a reference copy of the document with all sections, see nature.com/documents/nr-reporting-summary-flat.pdf

Life sciences study design

All studies must disclose on these points even when the disclosure is negative.

Sample size

180 women with early and locally advanced breast cancer planned to undergo neoadjuvant treatment were prospectively enrolled the molecular profiling study described (TransNEO). Of these, 12 were excluded and not sequenced (reasons: no research biopsy taken (n=6), co-diagnosis of metastatic disease (n=3), recruited to early stage clinical trials (n=2), died early during therapy (n=1)). Tumours from the 168 remaining women were molecularly profiled, of which 155 had associations with RCB and received adequate therapy exposure (defined as more than 1 cycle of chemotherapy and, if HER2+, more than 1 cycle of targeted therapy). This is summarised in Extended Data Figure 1 and in the Methods section.

For the validation dataset, sequenced cases within the control arm of the ARTemis trial (n=38) and cases within the PBCP study (n=37) that received neoadjuvant therapy and had DNA, RNA, and digital pathology data were used for validation (summarised in Extended Data Figure 1).

Data exclusions

To determine associations between response, only cases which had molecular/digital pathology data and received more than 1 cycle of chemotherapy and, if HER2+, received more than one cycle of targeted therapy were included (n=155 as described in Extended Data Figure 1 and Methods). These exclusion criteria were pre-established prior to commencing analysis to ensure that associations with response were only derived using data from patients treated with adequate therapy exposure (defined as more than one cycle of therapy).

Replication

The findings were validated in an independent dataset comprising 75 cases with DNA, RNA and digital pathology data.

Randomization

Randomization not applicable - all cases were treated with standard of care therapy regimens.

Blinding

Blinding not applicable - no group allocations.

Reporting for specific materials, systems and methods

We require information from authors about some types of materials, experimental systems and methods used in many studies. Here, indicate whether each material, system or method listed is relevant to your study. If you are not sure if a list item applies to your research, read the appropriate section before selecting a response.

Materials & experimental systems

n/a	Involved in the study	
☒	☐	Antibodies
☒	☐	Eukaryotic cell lines
☒	☐	Palaeontology and archaeology
☒	☐	Animals and other organisms
☐	☒	Human research participants
☐	☒	Clinical data
☒	☐	Dual use research of concern

Methods

n/a	Involved in the study	
☒	☐	ChIP-seq
☒	☐	Flow cytometry
☒	☐	MRI-based neuroimaging

Human research participants

Policy information about studies involving human research participants

Population characteristics	All participants within the TransNEO and PBCP studies were women diagnosed with early/locally advanced breast cancer and treated with neoadjuvant chemotherapy (and anti-HER2 therapy if HER2+) between 2013-2018. Participant characteristics are included within Supplementary data table 1.<br><br>The population characteristics of the patients used in the control arm of the ARTemis Study are described in Earl, H. M. et al. Efficacy of neoadjuvant bevacizumab added to docetaxel followed by fluorouracil, epirubicin, and cyclophosphamide, for women with HER2-negative early breast cancer (ARTemis): an open-label, randomised, phase 3 trial. Lancet. Oncol. 16, 656–66 (2015). Link to article: https://doi.org/10.1016/S1470-2045(15)70137-3
Recruitment	Within the TransNEO and PBCP studies, all women with early/locally advanced breast cancer presenting to Cambridge University Hospitals NHS Foundation Trust and planned to undergo pre-operative chemotherapy were approached by the Cambridge Breast Cancer Unit research team and offered participation within the study.<br><br>Inclusion criteria included:<br>1. Patient with histological diagnosis of invasive breast cancer<br>2. Patient receiving neoadjuvant therapy (chemotherapy and/or hormonal therapy)<br>3. Able to give informed consent<br>4. ECOG 0-2<br><br>In the ARTemis trial, key inclusion and exclusion criteria are available at https://www.clinicaltrialsregister.eu/ctr-search/search?query=2008-002322-11 and the trial description and results have been previously published https://doi.org/10.1016/S1470-2045(15)70137-3<br><br>There is no selection bias within this study: any patient identified in standard of care clinical practice to benefit from neoadjuvant therapy was approached to take part in the study, and all those who consented and donated tumour tissue were included in the study if they received more than one cycle of therapy and response assessment was available post therapy (Extended data figure 1).
Ethics oversight	East of England Research Ethics Committee: 12/EE/0484 (TransNEO), 18/EE/0251 (PBCP)<br>South East Research Ethics Committee: 08/H1102/104 (ARTemis)

Note that full information on the approval of the study protocol must also be provided in the manuscript.

Clinical data

Policy information about clinical studies

All manuscripts should comply with the ICMJEguidelines for publication of clinical research and a completedCONSORT checklist must be included with all submissions.

Clinical trial registration	ARTemis clinical trial: EudraCT Number 2008-002322-11, UK South East REC Number: 08/H1102/104, https://www.clinicaltrialsregister.eu/ctr-search/search?query=2008-002322-11
Study protocol	ARTemis clinical trial protocols:<br>https://www.clinicaltrialsregister.eu/ctr-search/trial/2008-002322-11/GB<br>https://warwick.ac.uk/fac/sci/med/research/ctu/trials/cancer/artemis/
Data collection	The ARTemis clinical trial collected data recruited women with early invasive breast cancer (radiological tumour size >20 mm, with or without axillary involvement), at 66 centres in the UK between May 7, 2009, and Jan 9, 2013. Full details of the trial have been published and are available within the supplementary material of the trial publication in Lancet Oncology: https://doi.org/10.1016/S1470-2045(15)70137-3
Outcomes	In the ARTemis trial, the primary endpoint was defined as complete pathological response rates after neo-adjuvant chemotherapy defined as no residual invasive carcinoma within the breast (DCIS permitted) AND no evidence of metastatic disease within the lymph nodes. The secondary endpoints were:<br>1. Disease-Free Survival<br>2. Overall Survival<br>3. Complete pathological response rates rate in the breast alone<br>4. Radiological (ultrasound) response after 3 and after 6 cycles of chemotherapy. Rate of breast conservation<br>Toxicities, including in particular cardiac safety and surgical complications (wound healing, bleeding, and thrombosis).<br><br>The results and assessment of these endpoints have already been published in Lancet Oncology: https://doi.org/10.1016/S1470-2045(15)70137-3
